# Mechanism of Activation of AMPK and Upregulation of OGG1 by Rapamycin in Cancer Cells

**DOI:** 10.18632/oncotarget.381

**Published:** 2011-12-21

**Authors:** Samy L. Habib

**Affiliations:** The University of Texas Health Science Center, Department of Cellular and Structural Biology

AMPK is a physiological cellular energy sensor that is activated by phosphorylation at Thr^172^ in response to changes in cellular ATP levels. AMPK has been recognized as an important upstream signaling intermediate intimately involved in the regulation of the mTOR pathway [1]. AMPK responds to energy stress by suppressing cell growth and biosynthetic processes, in part through its inhibition of the rapamycin-sensitive mTOR (mTORC1) pathway. In addition, AMPK directly phosphorylates the mTOR binding partner raptor on two well-conserved serine residues, and this phosphorylation induces 14-3-3 binding to raptor. The phosphorylation of raptor by AMPK is required for the inhibition of mTORC1 and cell cycle arrest induced by energy stress. Recent studies show that raptor was identified as a direct substrate of the AMPK. AMPK phosphorylates raptor on Ser^722^/Ser^792^ which it is essential for inhibition of the raptor-containing mTOR complex I (mTORCI). Moreover, AMPK is activated by the adenosine analogue, 5-aminoimidazole-4-carboxamide (AICA)-riboside (AICAR) and metformin. These drugs have been used to inhibit the growth and survival of breast cancer and glioblastomas cells [2].

mTOR is a large protein kinase with two different complexes. One complex contains mTOR, GβL and raptor, which is a target of rapamycin [3]. The other complex, insensitive to rapamycin, includes mTOR, GβL, Sin1, and rictor. The mTOR-rictor complex phosphorylates Ser^473^ of Akt/PKB, which is essential for full Akt/PKB activation. We have evidence in TSC-deficient proximal tubular cells accumulates significant amounts of ROS compared to wild type cells (data not shown). Accumulation of ROS is associated with activation/phosphorylation of Akt at Ser^473^, inactivation/phosphorylation of tuberin at Thr^1462^ and activation mTOR/rictor complex II [3]. In addition, Akt can be activated through the feedback mechanism of mTOR-rictor complex activation. Additional evidence indicates that rictor is phosphorylated at Thr^1135^ by p70S6K, which negatively regulates mTORC2 protein complex as part of a negative feedback mechanism controlling Akt activity [4].

Targeting mTOR is emerging as an important approach in cancer therapeutics [3]. Early clinical trials show that tuberous sclerosis complex (TSC) and Van Hippel-Lindau (VHL)-related kidney tumors regress in response to treatment with the mTOR inhibitor, rapamycin [5]. Treatment of TSC patients with rapamycin resulted in reduction in angiomyolipoma volume by nearly 50% [5]. mTOR serves a critical role in the regulation of the translational machinery and, in doing so, affects cellular responses to growth, proliferation, and differentiation, all of which are abnormally manifested in TSC lesions. TSC inhibits Rheb-GTP by promoting conversion to Rheb-GDP to block mTOR activity [6]. Although rapamycin can effectively inhibit mTOR activity and decrease kidney tumor size, the mechanisms involved are not fully understood On the other hand, a deficiency in DNA repair can lead to accumulated multiple gene mutations and result in tumors development [7]. Many of these mutations can occur as a result of irreparable or incompletely repaired genomic DNA, due to deficiencies in DNA repair enzymes such as 8-oxoG-DNA glycosylase (OGG1). Loss of OGG1 has major consequences in multistep carcinogenesis in the kidney and other organs [7]. Decreased OGG1 expression and activity leads to a mutator phenotype, with faulty repair of oxidized DNA lesions, and an accumulation of the oxidized DNA product (8-oxodG).

**Figure 1:**
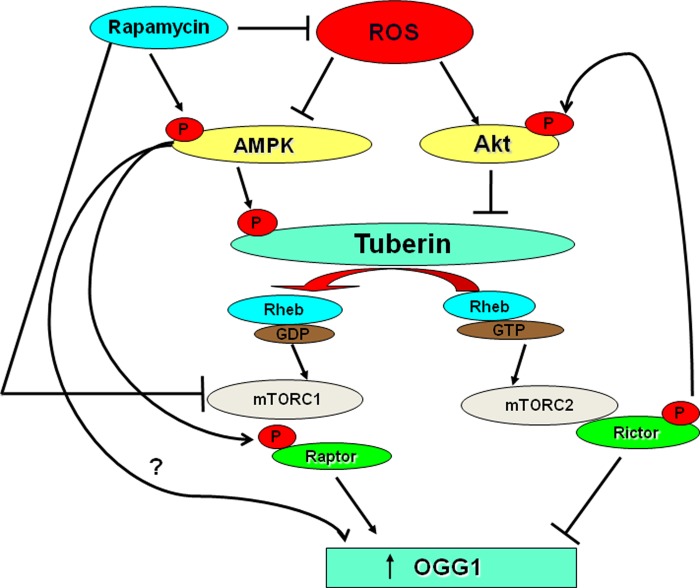
Rapamycin blocks ROS generation and directly or/and indirectly activates AMPK Activation of AMPK by rapamycin may impact directly on OGG1 activation or indirectly through blocking the activity of mTOR.

Upregulation of OGG1 was detected in several cancer cells treated with AMPK activator, AICAR [8]. Activation of AMPK resulted in activation/phosphorylation of TSC at Thr^1387^ and suppressed the mTOR activity. Moreover, rapamycin treatment led to a robust phosphorylation and activation of AMPK and ACC to a degree similar to that observed with AICAR stimulation in TSC2^+/−^ treated with HG further supporting the importance of rapamycin as an activator of AMPK. In addition, treatment of TSC-deficient mice with rapamycin showed inhibition of mTORC1 through increasing AMPK phosphorylation at Thr^172^ and it is downstream, ACC at Ser^79^ and accompanied by increased OGG1 activity [8]. Although lower concentrations of rapamycin appeared to inhibit mTOR activation in cancer cells, higher concentrations were required to increase the protein and promoter activity of OGG1, suggesting that high concentrations of rapamycin may be required to activates other kinases such as AMPK in addition to blocking mTOR activity [8]. In support of this hypothesis, rapamycin increases phosphorylation of AMPK, an upstream signaling intermediate in the mTOR pathway in different cancer cells. Moreover, rapamycin promotes AMPK activity as well as evidenced by increasing ACC phosphorylation. Activation of AMPK by rapamycin results in the inhibition of mTOR activity and increased the protein expression and promoter activity of OGG1 in proximal tubular cells. Moreover, activation of AMPK by AICAR resulted in inhibition of mTOR and increases the promoter activity and protein expression of OGG1 in tuberin-deficient and VHL-deficient cells [8]. Since AICAR increased the protein expression and the promoter activity of OGG1 in TSC2-null cells, it opens the possibility that induction of OGG1 by AMPK is through a pathway independent of TSC2. Thus AMPK may inhibits mTOR without activates of TSC2 and augment OGG1 expression, or, AMPK may induce OGG1 expression through unknown mTOR-independent mechanism (Figure 1). These data strongly suggest that rapamycin may acts as a direct activator of AMPK to phosphorylate raptor at Ser^722^/Ser^792^ and inhibit mTOR that lead to activate the DNA repair enzyme OGG1 in cancer cells.
